# Spontaneous Retroperitoneal Haematoma due to Polyarteritis Nodosa: Report of a Case and Literature Review

**DOI:** 10.1155/2016/7592563

**Published:** 2016-01-18

**Authors:** Emrah Simsek, Hasan Yilmaz, Kerem Teke, Ali Kemal Uslubas, Mustafa Yuksekkaya

**Affiliations:** Department of Urology, Kocaeli University Medical Faculty Hospital, 41380 Kocaeli, Turkey

## Abstract

Retroperitoneal haematoma is a rare clinical entity with variable etiology. It can happen spontaneously, without any obvious precipitating factors, the so-called spontaneous retroperitoneal haematoma. There is no general consensus as to the best management plan for patients with retroperitoneal haematoma. Polyarteritis nodosa (PAN) is a rare cause of retroperitoneal haematoma. Here we report relationship between PAN and retroperitoneal haematoma and treatment approaches. However, an accepted and clearly defined treatment has not been established due to its rarity.

## 1. Introduction

PAN is a systemic, necrotizing vasculitis affecting medium-sized and small arteries. It is characterized by segmental necrosis leading to aneurysm formation. Kidney involvement in PAN is seen in 70–80% of the patients and it may rarely be complicated by aneurismal rupture leading to subcapsular and perirenal haematomas [1]. Perirenal haematoma as the presenting feature of PAN can be diagnostically challenging, leading to a delay in recognition and treatment. We report a case of spontaneous perirenal haematoma in a young patient who had nonspecific abdominal pain. The patient received conservative treatment without surgical intervention and had an uneventful recovery.

## 2. Case Report

**Figure 1:**
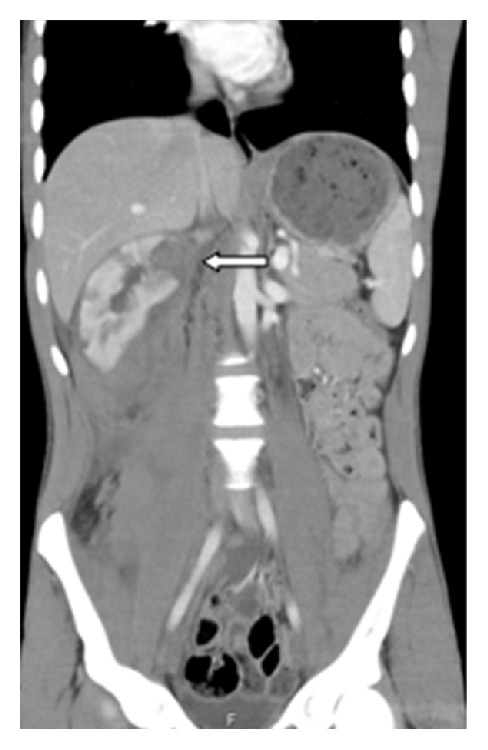
Contrast enhanced computed tomography of the patient where multiple infarcts can be seen in right kidney (arrows).

**Figure 2:**
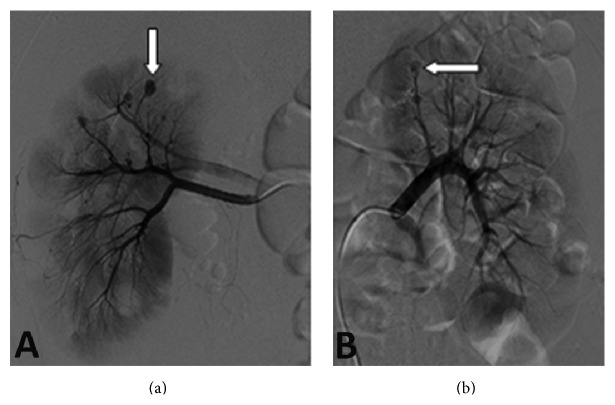
Angiographic studies of the vasculature of the right kidney, left kidney. An angiogram of the vasculature of the right kidney (a) reveals multiple small aneurysms; an angiogram of the vasculature of the left kidney (b) shows many small aneurysms.

A 24-year-old man was referred to emergency service because of nontraumatic excruciating abdominal pain. Assessment of patient's history revealed that there have been intermittent abdominal pain, fever, loss of appetite, 10 kg weight loss, weakness, myalgia, and arthralgia of small joints in the last few months. He had no history of bleeding diathesis, or anticoagulant therapy. On physical examination, the patient appeared uncomfortable. His temperature was 37.4°C, his pulse was regular at 80 beats per minute, and his blood pressure was 110/60 mmHg. He had left costovertebral angle tenderness. There were no rushes and skin lesions, the testes were nontender, and genital system looked normal. The results of laboratory examinations revealed leukocytosis of 23,9 (4,6–10,2), anemia with hemoglobin level of 9,6 (12,2–18,1), and elevated sedimentation rate of 70 (0–15). Renal function tests and other biochemical parameters were unremarkable. The urinalysis showed proteinuria (+++) and haematuria (+++). Hepatitis B surface antigen, antinuclear antibody, and antineutrophil cytoplasmic antibodies were negative. A contrast enhanced computed tomography of the abdomen showed multiple small wedge-shaped less-enhanced areas (Figure 1) with suspicion of intrarenal hemorrhage, hemorrhagic cyst rupture, or right perirenal haematoma. The patient was started on broad spectrum antibiotics after blood and urine cultures were obtained. His hemoglobin initially decreased to 6.5 g/dL and then stabilized at 10.0 g/dL after transfusion of 7 units of packed red blood cells. His blood pressure levels elevated about 160/100 mmHg on follow-up and stabilized using ACE inhibitor. When the patient became hemodynamically stable, a selective renal arteriogram was performed (Figure 2) which demonstrated multiple small aneurysms in the segmental and interlobar arteries of both kidneys but extravasation was not noted. Based on the clinical picture and multiple aneurysms on arteriogram, a diagnosis of polyarteritis nodosa was made in accordance with the American College of Rheumatology 1990 criteria by rheumatologist. The patient underwent right ureteral stent placement due to the renal colic and hydronephrosis. He was given intravenous methyl prednisolone 60 mg/day and intravenous pulse methyl prednisolone (1 day) followed by intravenous cyclophosphamide. After five pulses of intravenous cyclophosphamide, it was shifted to azathioprine along with low-dose steroids. He remained asymptomatic with no recurrence of symptoms at 6 months of follow-up and his blood pressure was normal with amlodipine and ramipril.

## 3. Discussion

Spontaneous retroperitoneal hemorrhage also known as Wunderlich syndrome (WS) is a rare but potentially life threatening entity characterized by acute onset of nontraumatic subcapsular and perirenal haematomas. Spontaneous perirenal bleeding secondary to PAN was first described by Schmidt in 1908. It is classically characterized by Lenk's triad: acute flank pain, abdominal tenderness, and signs of internal bleeding. A wide spectrum of neoplastic and nonneoplastic renal pathologies may result in WS. Zhang et al. reported on 135 such cases in their meta-analysis. Benign and malignant neoplasms accounted for the majority of cases (61%), with the most common being angiomyolipoma and renal cell carcinoma. Vascular causes were the next major cause with PAN accounting for 28 cases (17%) [2]. Other causes of WS include arteriovenous malformations, venous thrombosis, cystic renal diseases, renal calculi, nephritis, and coagulation disorders. PAN is a necrotizing systemic inflammatory vasculitis with a peak incidence between the age of 40 and 60 years. It most commonly affects the kidneys and skin. Aneurysms can develop due to segmental necrosis, which correlate with the severity of the illness and may cause the development of thrombosis, rupture, and hemorrhage [3]. Spontaneous renal hemorrhage or rupture is a rare complication. The prognosis for patients with untreated PAN is merciless with a 1-year survival rate of 50%; death is often a consequence of renal failure, myocardial infarction, or stroke [4]. With appropriate therapy generally based on prednisone and cyclophosphamide, the 5-year survival rate is approximately 80% [5]. Given the poor prognosis associated with untreated PAN and the substantial improvement with appropriate treatment, it should be kept in mind in the differential diagnosis in order to minimize morbidity and the risk of death. A ruptured aneurysm resulting in a huge perirenal haematoma usually necessitates an urgent surgery.

Sometimes urgent nephrectomy is life-saving [6]. But selected cases can be treated conservatively as in our case. Also therapeutic arterial embolization is becoming an alternative to surgery in patients with hemorrhage from ruptured aneurysm, especially those in critical condition [7]. However, in this case, our patient required neither embolization nor nephrectomy. In our patient, an acute drop in hemoglobin levels occurred after a hypertensive attack. So hypertension should be treated aggressively. In conclusion, although we acknowledge that treatment options should be individualized and where necessary surgery should be performed to save the patient life, we still recommend careful diagnosis and observation to enable a case of retroperitoneal hemorrhage that spontaneously ceases to bleed to be managed conservatively. In this case report, we show conservative treatment of retroperitoneal haematoma because PAN is a viable option in addition to invasive procedures.
